# Utilization of triangle nanosilver to prepare spherical nanosilver and quantitatively detect trace titanium by SERS

**DOI:** 10.1186/1556-276X-9-663

**Published:** 2014-12-10

**Authors:** Qingye Liu, Guiqing Wen, Xinghui Zhang, Aihui Liang, Zhiliang Jiang

**Affiliations:** 1 Key Laboratory of Ecology of Rare and Endangered Species and Environmental Protection of Ministry Education, Guangxi Key Laboratory of Environmental Pollution Control Theory and Technology, Guangxi Normal University, Guilin 541004, China

**Keywords:** Blue triangle nanosilver, Spherical nanosilver, Safranine T, SERS quantitative, Ti

## Abstract

The blue triangle nanosilver (BAgNP) sol was prepared by the two reducers of NaBH_4_ and H_2_O_2_. Using BAgNP as the precursor, a small spherical nanosilver (AgNP) sol in yellow was synthesized by addition of suitable amounts of *X*^−^ (*X* = Cl, Br, and I). The oxidization process of BAgNP to AgNP was studied in detail by resonance Rayleigh scattering (RRS), surface-enhanced Raman scattering (SERS), laser scattering, surface plasmon resonance (SPR) absorption, and microscope techniques. It has been observed that NaCl accelerated the oxidizing BAgNP to form AgNP, and an oxidizing mechanism and quasi-nanograting Raman-scattering enhanced mechanism were developed to explain the phenomena. Using the BAgNP sol as substrate and based on the catalysis of Ti(IV) on the BrO_3_^−^ oxidizing safranine T (ST) molecular probe with a strong SERS peak at 1,535 cm^−1^, a new catalytic SERS quantitative method was developed for the determination of 1.0 to 100 ng/mL Ti, with a detection limit of 0.4 ng/mL.

## Background

Metal nanoparticles, especially nanogold and nanosilver, are of novel physical and chemical properties that become one of the hot spots in physics, chemistry, materials, and sensors. Nanogold sol has good biocompatibility and stability and excellent optical and catalytic properties and has been used widely in several fields. Comparing to nanogold, the cost of nanosilver is lower, the mol absorption coefficient is higher, and the optical property is more excellent such as a very high surface-enhanced Raman scattering (SERS) effect and very low mole absorption coefficient of its aggregations. In addition, its studies and applications are less than the nanogold. These are interesting to people to study the nanosilver preparation, property, and applications [1-3].

It is significant to prepare nanosilver sol because it exhibits novel optical properties such as different color and high SERS activity and can be used as a biochemical label and nanocatalyst in liquid phase synthesis. In general, nanosilver sols were prepared by citrate heating and NaBH_4_ procedures. Lee et al. [4] used NaBH_4_ as the reducer to prepare a brown nanosilver sol with a surface plasmon resonance (SPR) peak at 400 nm. Using citrate as the reducer, an unstable kelly nanosilver sol was obtained with a SPR peak at 420 nm. Munro et al [5] improved the citrate procedure to prepare stable nanosilver sol by addition of a stable reagent. However, the addition of the stable reagent made the procedure complicated, and a serious problem may be caused in that it interfered with the subsequent study. For example, the stable reagent may affect the nanosilver functionalizing and restrain the optical property. A stable blue triangle nanosilver sol was prepared by NaBH_4_ and H_2_O_2_ reducers [6,7], using polyvinylpyrrolidone (PVP) as the stable reagent. However, the PVP strongly restrains the SERS effect that decreased SERS sensitivity. Thus, it is important to prepare stable, simple, highly SERS-active nanosilver sol without a restraining stabilizer. To our best knowledge, there are no reports that used big triangle nanosilver to prepare small spherical nanosilver and to determine trace Ti by SERS technique. In this article, the blue triangle nanosilver (BAgNP)-NaCl system was studied firstly by resonance Rayleigh scattering (RRS) [8-10] and SERS [11,12] spectral techniques. A simple and rapid preparation procedure for yellow nanosilvers (AgNPs) was developed using BAgNPs as the precursor. In addition, titanium is a necessary trace element for organisms that enhanced the immune function and stimulated plant growth. Therefore, it is important to develop a simple, rapid, sensitive, and selective method for the determination of trace Ti in plant and foods. At present, several methods including atomic, molecular, and mass spectrometry have been reported for the analysis of Ti [13,14]. However, there are no SERS methods with rapidity, high sensitivity, and selectivity for quantitative analysis of Ti in foods such as tea. Thus, a new catalytic SERS method was developed for the quantitative analysis of trace Ti, based on its catalysis of BrO_3_^−^ oxidization of safranine T (ST) that can be utilized to amplify the analytical signal, and using ST as the SERS molecular probe in the BAgNP sol substrate that formed highly SERS-active AgNP/AgCl composite aggregations in the presence of NaCl.

## Methods

### Materials

Stock standard solutions of 1.0 × 10^−3^ mol/L AgNO_3_, 1.0% (*W*/*V*) trisodium citrate, 0.05 mol/L NaCl, 30% H_2_O_2_, freshly prepared 0.05% NaBH_4_, 10 mmol/L KBrO_3_, 1.0 × 10^−5^ mol/L ST, 1.0 mol/L H_3_PO_4_, and 1.00 mmol/L Ti(IV) were prepared. A 1.0 × 10^−4^ mol/L BAgNP sol was prepared as follows: into a triangle flask containing about 40 mL water, 500 μL 1.0 × 10^−2^ mol/L AgNO_3_, 1.5 mL 6.0 × 10^−2^ mol/L trisodium citrate, 200 μL 0.1 mol/L NaBH_4_, and 120 μL 30% H_2_O_2_ were added in turn with constant stirring for 15 min and diluted to 50 mL to obtain the BAgNP sol. To obtain BAgNP sol without H_2_O_2_ (hBAgNP), the BAgNP sol could be heated at 60°C for 15 min to get rid of the excess H_2_O_2_, and the solution was also in blue. The stable AgNP sol in yellow was prepared by mixing 10 mL 1.0 × 10^−4^ mol/L BAgNP with 30 μL 0.50 mol/L NaCl or 10 μL 0.005 mol/L NaBr solutions. All reagents were of analytical grade and the water was highly pure sub-boiling water.

### Apparatus and measurements

A model F-7000 fluorescence spectrophotometer (Hitachi Company, Chiyoda-ku, Japan) was used to record the RRS intensity, and the RRS spectra were recorded by means of synchronous scanning excited wavelength *λ*_ex_ and emission wavelength *λ*_em_ (*λ*_ex_ − *λ*_em_ = Δ*λ* = 0). A model DXR smart Raman spectrometer (Thermo Fisher Scientific Co., Ltd., Waltham, MA, USA) was used to record the SERS spectra and the intensity using a laser wavelength of 633 nm, power of 2.0 mW, and collection time of 2.0 s. A model of TU-1901 double beam UV-vis spectrophotometer (Beijing Purkinje General Instrument Co. Ltd., Beijing, China), a model of JEM-2100 F field emission transmission electron microscope (Electronic Stock Limited Company, Tokyo, Japan), and a model of nanoparticle and Zeta potential analyzer (Malvern Instruments Ltd., Malvern, England) were used.

#### Procedure for preparation and spectral characterization of AgNP

Into a test tube, 1.0 mL 1.85 × 10^−4^ mol/L BAgNP solution and certain amounts of *X*^−^ were added, diluted to 2 mL with water, and mixed well to obtain the AgNP sol. The RRS spectra and the intensity (*I*) were recorded by a fluorescence spectrophotometer with the synchronous scanning technique (*λ*_ex_ − *λ*_em_ = Δ*λ* = 0). A blank (*I*_0_) without *X*^−^ was recorded, and the value of Δ*I = I − I*_0_ was calculated. Meanwhile, the SPR absorption spectra were also recorded by spectrophotometer. If the ST molecular probes were added after the addition of *X*^−^, the SERS spectra and the intensity were recorded by the laser Raman spectrometer.

#### Procedure for SERS detection of Ti

A solution of 100 μL 1.0 × 10^−5^ mol/L ST, 250 μL 10 mmol/L KBrO_3_, and 100 μL 1 mol/L H_2_SO_4_ and a certain amount of Ti(IV) solution were added into a 5-mL marked test tube, diluted to 1.0 mL and mixed well. The mixture was placed in 60°C for 10 min and cooled with tap water, and 120 μL 1.0 mol/L NaCl and 500 μL 100 μmol/L BAgNP solutions were added, diluted to 2.0 mL, and mixed well. Then, the mixture was transferred into a 1-cm quartz cell. The SERS spectrum and the SERS intensity at 1,535 cm^−1^ (*I*_1,535cm-1_) were recorded. Meanwhile, a reagent blank (*I*_1,535cm-1_)_0_ without Ti(IV) was recorded, and a value of Δ*I* = (*I*_1,535cm-1_)_0_ − *I*_1,535cm-1_ was calculated.

## Results and discussion

### Principle for SERS detection of Ti

**Figure 1:**
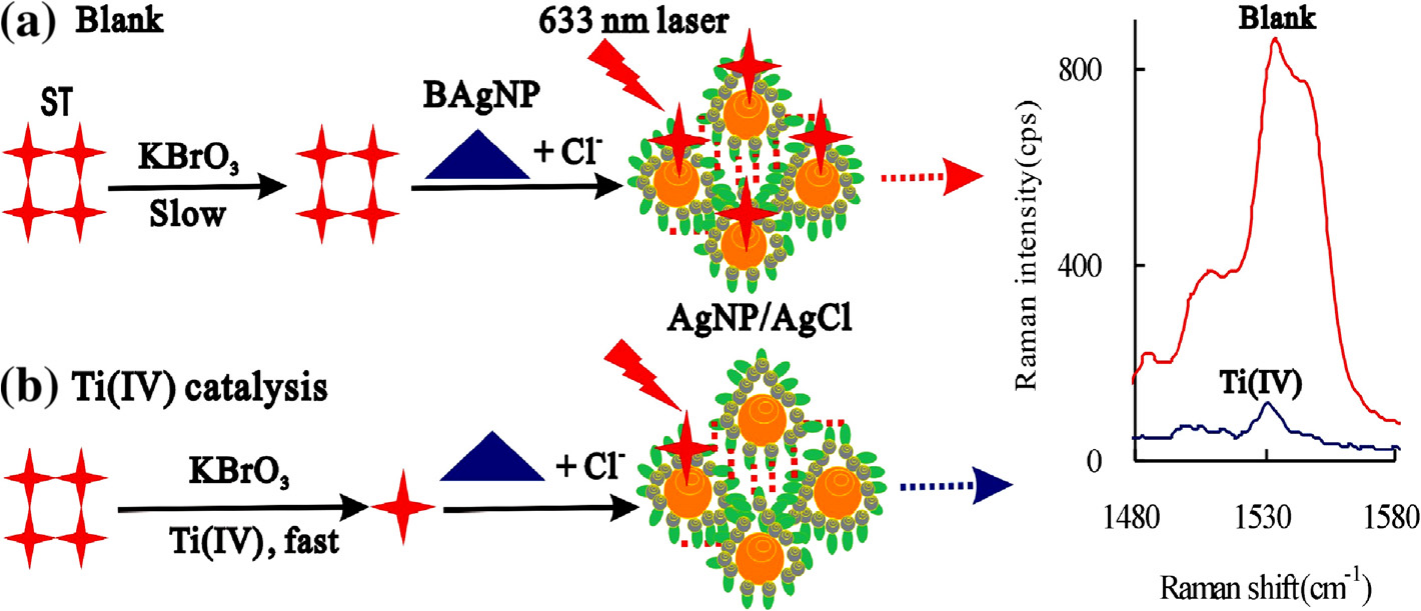
**Principle for SERS detection of Ti, based on its catalysis of KBrO**_**3**_**-ST reaction. (a)** Blank: the uncatalytic reaction of KBrO_3_-ST was slow, and the SERS peak at 1535 cm^−1^ was strong; **(b)** Ti(IV) catalysis: the catalytic reaction was fast, and the SERS peak was weak.

In the as-prepared BAgNP sol substrate and in the presence of NaCl, ST molecules adsorbed on the surfaces of AgNP/AgCl aggregation by hydrophobic and intermolecular forces and exhibited a sensitive SERS peak at 1535 cm^−1^ that could be used as a SERS probe to monitor the ST concentration changes in the catalytic reaction system. In the absence of a Ti(IV) catalyst, the redox between BrO_3_^−^ and ST was very slow that a strong SERS peak appeared at 1535 cm^−1^. When the Ti(IV) catalyst increased, the redox enhanced and the ST concentration decreased that caused the SERS peak to decrease linearly. Thus, a new catalytic SERS quantitative analysis method was developed for trace titanium, based on the catalysis of Ti(IV) on the ST-BrO_3_^−^ reaction as in Figure 1.

### Preparation of nanosilver sols

The conditions for preparing BAgNP sol, including the precursor AgNO_3_, stabilizer sodium citrate, reducer of NaBH_4_ and H_2_O_2_, and reaction temperature and time, were considered. Those conditions as in the ‘Methods’ section were selected to prepare stable BAgNP and hBAgNP sols. Using the BAgNP or hBAgNP sol as the precursor, the preparing conditions of yellow nanosilver such as NaX and H_2_O_2_ were also examined. Without addition of H_2_O_2_, there are still micro-amounts of H_2_O_2_ in the BAgNP sol that come from the preparation process. Upon the addition of NaCl in the range of 5 × 10^−4^ to 100 × 10^−4^ mol/L, the color changed from blue to yellow, the characteristic SPR absorption peak shifts from 550 to 395 nm, and the RRS intensities enhanced due to the formation of rigid AgNP and loose AgNP/AgCl particles. The NaCl concentration increased continuously, and the color is gray due to the aggregation of AgNPs. In the absence of NaCl, the residual H_2_O_2_ in the BAgNP sol cannot oxidize the BAgNP. When the H_2_O_2_-added concentration is higher than 0.003%, the blue color comes out immediately. In the presence of NaCl, as low as 0.0001% H_2_O_2_ also oxidizes BAgNP to form Ag^+^. In short, even with no addition of H_2_O_2_, the stable yellow nanosilver sol can be obtained by mixing BAgNP sol and a suitable *X*^−^.

### Characterization of nanosilver

**Figure 2:**
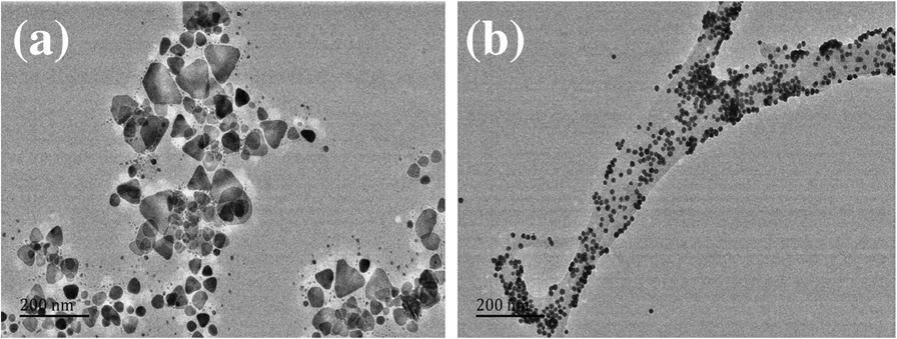
**TEMs of blue triangle nanosilvers and spherical nanosilvers. (a)** The 1.0 × 10^−5^ mol/L BAgNP was prepared by the two reducers of NaBH_4_ and H_2_O_2_; **(b)** the spherical nanosilvers were obtained by mixing 1.0 × 10^−5^ mol/L BAgNP and 15 × 10^−4^ mol/L NaCl.

**Figure 3:**
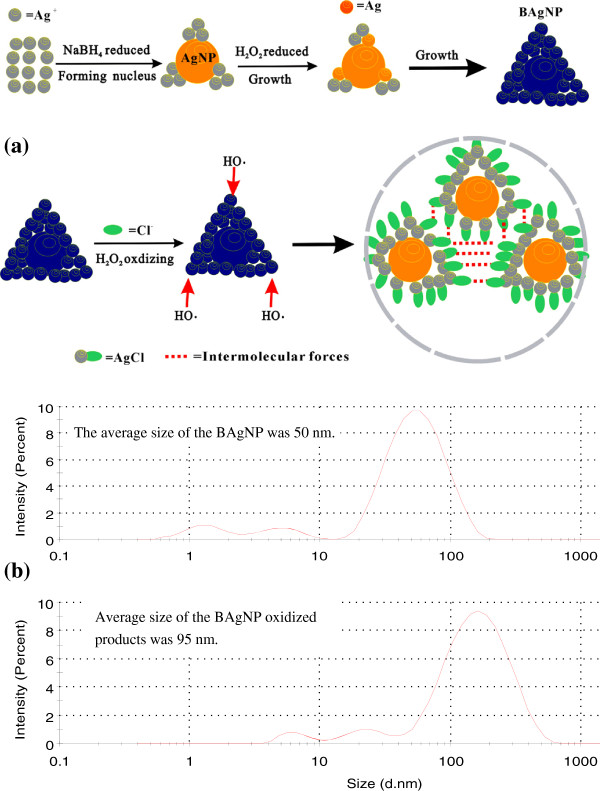
**The formation and oxidization processes of BAgNPs and their size distribution. (a)** BAgNPs were prepared by the two reducers of NaBH_4_ and H_2_O_2_, and they could be oxidized to form big AgNP/AgCl particles by the excess H_2_O_2_, in the existence of NaCl; **(b)** the size distribution of BAgNP was in the range of 0.5 to 200 nm with an average size of 50 nm, and the size distribution of the spherical nanosilver sol was in the range of 2 to 500 nm with an average size of 95 nm.

In the presence of citrate, Ag^+^ reduced rapidly by NaBH_4_ to form a nanosilver nucleus such that the absorbed Ag^+^ can be reduced by H_2_O_2_ and growth to BAgNP with the side length between 20 and 80 nm (Figure 2a) that the average size of 50 nm was recorded by laser scattering technique (Figure 3). Upon addition of NaCl into the BAgNP, the Cl^−^ ions were absorbed on the surface of BAgNP in a triangle, and the remnant H_2_O_2_ can be also absorbed on the surface. The Ag atoms on the angle of BAgNP, that is the (111) crystal surface, have strong activity, in which HO**·**radicals produced from H_2_O_2_ catalytic decompose and quickly oxidized the Ag atoms on the angle to produce Ag^+^ that combined with Cl^−^ to form strongly hydrophobic AgCl molecules, and the Ag atoms on other places were oxidized slowly. If there are Cl^−^ ions, the oxidization is over. Thus, yellow spherical AgNPs with a size of 15 nm (Figure 2b) were obtained by addition of suitable amounts of NaCl in the BAgNPs. The atoms of the BAgNP surface can be oxidized asymmetrically to form Ag^+^ that reacts with the Cl^−^ to produce AgCl molecules. The AgCl molecules have strong intermolecular forces that make it together to form big AgNP/AgCl aggregates with an average size of 95 nm that was recorded by laser scattering technique (Figure 3b). Thus, the color is yellow and the RRS enhanced when NaCl was added (Figure 3). When the hBAgNP sols without H_2_O_2_ were substituted the BAgNP sol containing H_2_O_2_, no color change was observed. This indicated that the H_2_O_2_ is important to prepare the yellow nanosilver sol. Similar to Cl^−^, the blue BAgNP sol was changed to yellow when it mixed with Br^−^ or I^−^.

### SPR absorption spectra

**Figure 4:**
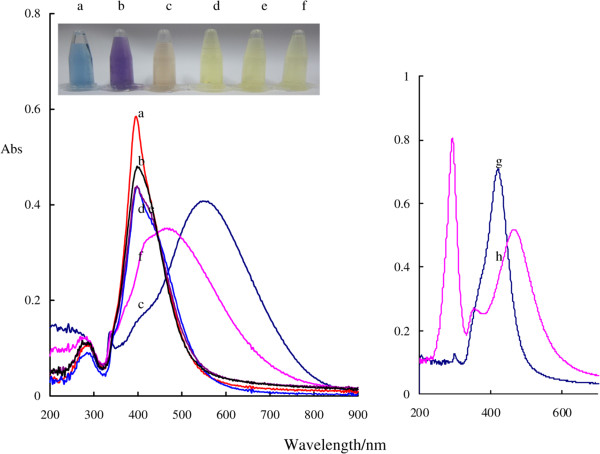
**Absorption spectra of the BAgNP-NaX sols in different colors. (a)** 5.0 × 10^−5^ mol/L BAgNP sol was in blue; **(b)** the BAgNP sol was mixed with 5.0 × 10^−4^ mol/L NaCl that showed a blue-violet color; **(c)** (a) + 10 × 10^−4^ mol/L NaCl solution in light yellow; **(d)** (a) + 15 × 10^−4^ mol/L NaCl solution in yellow; **(e)** (a) + 100 × 10^−4^ mol/L NaCl solution in yellow; **(f)** (a) + 250 × 10^−4^ mol/L NaCl solution in yellow; **(g)** 5.0 × 10^−5^ mol/L BAgNP + 2.5 × 10^−6^ mol/L NaBr solution in yellow; **(h)** 5.0 × 10^−5^ mol/L BAgNP + 40 × 10^−6^ mol/L KI solution in deep yellow.

Spherical AgNPs have the strongest SPR absorption peak near 400 nm that is an out-of-plane dipole SPR absorption peak, and triangle nanosilvers have three SPR absorption peaks. The BAgNPs exhibited a sharp SPR absorption peak at 340 nm that is corresponding to the out-of-plane quadrupole, a weak out-of-plane dipole SPR absorption peak at about 400 nm, and a wide peak at 550 nm ascribed to in-plane dipole (Figure 4(a)). When Cl^−^ increased, the color was changed from blue to blue-purple and yellow, the out-of-plane quadrupole peak disappeared at 340 nm, and the in-plane dipole peak at 550 nm shifted to violet and appeared at a SPR peak at 395 nm that indicated the existence of spherical nanosilvers in the system (Figure 4(d)). For the Br^−^ system, there is only one SPR absorption peak at 416 nm ascribed to the out-of-plane dipole of the spherical nanoparticle (Figure 4(g)). For the I^−^ system, there is a SPR absorption peak at 458 nm and an absorption peak at 292 nm ascribed to I^−^ ions (Figure 4(h)). Without addition of H_2_O_2_, the stable yellow nanosilver sol can be obtained by mixing BAgNP sol and a suitable *X*^−^.

### RRS spectra

**Figure 5:**
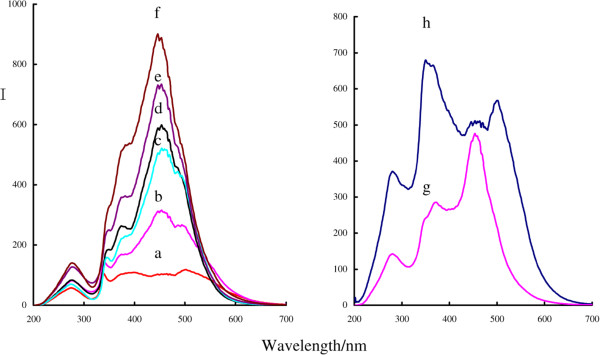
**RRS spectra of the BAgNP-NaX system. (a)** the 5.0 × 10^−5^ mol/L BAgNP sol exhibited a weak RRS signal at 457 nm; **(b)** the (a) + 5.0 × 10^−4^ mol/L NaCl system exhibited the strongest RRS peak at 457 nm; **(c)** the (a) + 10 × 10^−4^ mol/L NaCl system exhibited a strong RRS peak at 457 nm; **(d)** the (a) + 15 × 10^−4^ mol/L NaCl system exhibited the strongest RRS peak at 457 nm; **(e)** the a + 100 × 10^−4^ mol/L NaCl system exhibited the strongest RRS peak at 457 nm; **(f)** the (a) + 250 × 10^−4^ mol/L NaCl system exhibited the strongest RRS peak at 457 nm; **(g)** the 5.0 × 10^−5^ mol/L BAgNP + 2.5 × 10^−6^ mol/L NaBr system exhibited the strongest RRS peak at 456 nm; **(h)** the 5.0 × 10^−5^ mol/L BAgNP + 40 × 10^−6^ mol/L KI system exhibited the strongest RRS peak at 355 nm.

RRS was a simple and sensitive technique to detect nanoparticles and its aggregations. In general, the increased size and the aggregated nanoparticles all caused the RRS signal to enhance. The BAgNPs in sol are small sized and stable that exhibited weak RRS signal (Figure 5). With addition of NaCl, the angle Ag atoms of BAgNP were oxidized catalytically by the HO·from H_2_O_2_ to produce [Ag^+^]. The [Ag^+^] combined with Cl^−^ to form [AgCl] with strong hydrophobic property that was aggregated to larger AgNP/AgCl particles; the RRS peaks at 286, 340, 380, 457, and 500 nm are all enhanced, and the RRS peak at 457 nm is strongest. If the Br^−^ and I^−^ were substituted to Cl^−^, the Br^−^ and I^−^ systems exhibited three RRS peaks at 281, 375, and 456 nm and three peaks at 283, 355, and 504 nm (Figure 5(g),(h)). The enhanced RRS signals demonstrated that there are hydrophobic AgX molecules in the systems.

### SERS spectra

**Figure 6:**
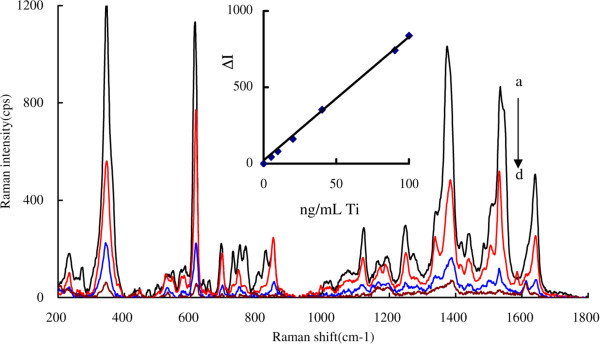
**SERS spectra of the Ti(IV)-KBrO**_**3**_**-ST catalytic reaction in the BAgNP sol substrate. (a)** 0.5 μmol/L ST +1.25 mmol/L KBrO_3_ + 50 mmol/L H_2_SO_4_ + 15 min +60 mmol/L NaCl +25 μmol/L AgNPB; **(b)** (a) + 40 ng/mL Ti; **(c)** (a) + 90 ng/mL Ti; **(d)** (a) + 100 ng/mL Ti. The inserted figure of working curve showed that the decreased SERS intensity at 1,535 cm^−1^ was linear to the Ti concentration in the range of 1.0 to 100 ng/mL.

SERS was a very sensitive technology for molecular detection, and it is particularly important to select a suitable SERS substrate. Noble metals such as silver are high SERS activity and commonly selected as nanosol substrate [15,16]. The spherical, triangle, rod, flower, and cap nanosilvers have been used as SERS substrates [17,18]. To prepare stable triangle nanosilver sol, PVP was selected as stable reagent but it restrained strongly the SERS activity that was confirmed by us. As a good SERS quantitative analysis nanosol, it would be of high SERS activity, good stability, good reproducibility, and low-cost reagents to being obtained easily. Without PVP, a good BAgNP sol was prepared by our research group using NaBH_4_ and H_2_O_2_ as reducers. In the BAgNP sol substrate and in the absence of NaCl as aggregated enhancement reagent, the SERS molecular probe of ST exhibited a very weak Raman signal due to rare AgNP/AgCl aggregations and the BAgNPs dispersing greatly in the solution system. In the presence of NaCl, there are AgCl molecules and AgNP/AgCl aggregations, in which spherical AgNPs were linked by means of strongly hydrophobic AgCl molecules to form the hemline and groove of grating that was called as quasi-nanograting [19,20]. The Raman scattering photons of ST molecules on the grating take place due to diffraction and resonance that caused the signal to be enhanced greatly. When NaCl increased, the five strong SERS peaks at 347, 614, 1,376, 1,535, and 1,644 cm^−1^ enhanced due to more AgNP/AgCl aggregations forming. This also demonstrated that there are AgNPs and AgCl molecules in the system. According to the references of [12,21], the peak at 347 cm^−1^ was ascribed to the C-C stretch vibration, the peak at 614 cm^−1^ was ascribed to the benzene in-plane, the peak at 1,376 cm^−1^ was ascribed to the C-N in-plane, the peak at 1,535 cm^−1^ was ascribed to the benzene ring stretch, and the peak 1,644 cm^−1^ was ascribed to the C = N stretch. Under the selected conditions for the catalytic analytical system, the decreased SERS intensity at 1,535 cm^−1^ was linear to the Ti concentration in the range of 1.0 to 100 ng/mL, and the peak at 1,535 cm^−1^ with best selectivity was selected for use (Figure 6).

### SERS quantitative analysis of Ti

The application of catalysis is a new way to amplify analytical signal in resonance Rayleigh scattering and SERS [22-24]. For example, a highly sensitive SERS method was reported for the determination of trace nitrite, based on nitrite catalyzing the bromate oxidization of rhodamine 6G probe [25]. ST is a kind of aromatic amine alkali industrial dye that has been used in spectrophotometry [26]. We have found that ST could be used as a SERS molecular probe in the BAgNP sol substrate for the determination of trace Ti, based on its catalytic effect on the BrO_3_^−^ oxidizing ST in H_2_SO_4_ medium at 60°C. Here, we report a new SERS method for detection of trace Ti(IV), based on its catalytic effect. The analytical conditions for the SERS quantitative analysis of Ti were examined. A 0.5 μmol/L ST, 1.25 mmol/L KBrO_3_, 50 mmol/L H_2_SO_4_, 60°C reaction for 10 min, 0.06 mol/L NaCl as aggregated enhancement reagent, 25 μmol/L BAgNP, and a reproducible peak at 1,535 cm^−1^, giving high sensitivity and good accuracy, were chosen for use. Under the selected conditions, the decreased SERS intensity at 1,535 cm^−1^ was linear to Ti concentration in the range of 1.0 to 100 ng/mL as in the inserted figure of Figure 6, with a regress equation of Δ*I* = 8.03C + 20, coefficient of *R*^2^ = 0.9977, and a detection limit of 0.4 ng/mL. The influence of foreign metal ions on the determination was examined within an error of ±10%. Results (Table 1) indicated that common metal ions do not interfere with the determination of 40 ng/mL Ti, and this method has good selectivity. The Ti in tea samples was determined by this SERS method. A 1.00-g tea samples were weighed into a beaker containing 20 mL of mixed acid (HNO_3_/HClO_4_ = 4/1 in volume ratio), heated to decompose and to near dry. The mixture was dissolved with 10 mL 0.10 mol/L H_2_SO_4_ solution and diluted to 25 mL with water. The sample solutions were used to determination of Ti content, with a relative standard deviation (RSD) of 3.5% to 5.3%. A recovery of 91.8% to 109% was obtained when a known Ti was added the samples (Table 2). The analytical results of the SERS method were in agreement with that of the atomic absorption spectrometry (AAS) [27], and this indicated that the catalytic SERS method was accurate.

**Table 1:** Effect of coexistent ions on the SERS quantitative analysis of 40 ng/mL Ti

**Coexistent substance**	**Tolerance limit(times)**	**Relative error (%)**	**Coexistent substance**	**Tolerance limit(times)**	**Relative error (%)**
Ca^2+^	400	4.1	Zn^2+^	100	3.8
Mg^2+^	400	3.9	Mn	100	4.6
Al^3+^	300	5.0	Cu^2+^	100	5.2
Cr^3+^	200	6.0	Cu^2+ a^	200	5.2
Pb^2+^	150	5.2	Fe^3+^	20	5.4
Ba^2+^	300	6.2	Fe^3+ a^	400	4.6
Co^2+^	100	2.9	Cd^2+^	400	4.3

^a^Containing 10 mg/mL hydroxylamine hydrochloride.

**Table 2:** Analytical results for Ti in tea samples using the catalytic SERS and AAS methods

**Sample**	**Single value (μg/g Ti)**	**Average (μg/g Ti)**	**RSD (%)**	**Added Ti (μg/g Ti)**	**Found Ti (μg/g Ti)**	**Recovery (%)**	**AAS (μg/g Ti)**
1	9.50, 9.85, 10.3, 9.40, 10.5	9.91	3.5	10.0	19.1	91.8	10.3
2	11.1, 11.0, 12.5, 11.8, 11.9	11.7	5.3	10.0	22.8	109	12.0
3	13.5, 14.3, 13.2, 14.2, 14.5	13.9	4.0	10.0	24.8	106	13.1

## Conclusions

In summary, a SERS-active and stable BAgNP sol was prepared by the NaBH_4_-H_2_O_2_ procedure without PVP surfactant. Using the BAgNPs as the precursor, a simple and fast procedure was developed for preparation of stable yellow nanosilver sol by mixing it with NaCl that can be used as a SERS sol substrate with strong SERS activity. The nanosilver sols and their oxidization were studied in detail, and the oxidization and quasi-nanograting enhanced mechanisms were proposed to explain the phenomena. Using BAgNP-NaCl as the SERS substrate and ST as the probe, a new catalytic SERS method was developed for determination of trace Ti in tea samples, with high sensitivity and selectivity.

## Competing interests

The authors declare that they have no competing interests.

## Authors’ contributions

QYL and GQW drafted the manuscript. QYL, GQW, and XHZ carried out the studies. AHL and ZLJ conceived the study and participated in its design and coordination and helped draft and revise the manuscript. All authors read and approved the final manuscript.
